# Atrio-Esophageal Fistula: A Rare Entity Complicating a Common Procedure

**DOI:** 10.1155/2023/3930221

**Published:** 2023-04-04

**Authors:** I. A. Sanoja

**Affiliations:** Oregon Health and Science University, Portland, OR, USA

## Abstract

A 66-year-old female with a history of radiofrequency ablation for atrial fibrillation presented with hematemesis and fever. A CT chest revealed an atrio-esophageal fistula (AEF) and a CT head showed bilateral septic emboli. Blood cultures were positive for *Streptococcus sanguinis*. She underwent primary repair of the atrial defect on cardiopulmonary bypass where a large atrial vegetation was retrieved, followed by a right thoracotomy with the closure of the esophageal defect the next day. She was discharged to a rehabilitation facility after 18 days of hospital stay with a 6 weeks antibiotics plan. The incidence of AEF following ablation procedures has been estimated at 0.01 to 0.04%, and the pathogenesis is linked to direct tissue and vagus nerve injury. The most common clinical findings are fever and neurologic deficits. CT chest is the best diagnostic modality. CT head might demonstrate embolic phenomena and TTE can show vegetation. Early surgical intervention, even in an unstable patient, is paramount for survival.

## 1. Introduction

Atrio-esophageal fistula (AEF) is a rarely seen but known complication of catheter ablations, with an estimated mortality of 40-100%. In this case, I describe the natural history of a patient presenting with AEF and summarize the pathogenesis, diagnostic strategies, and management options before discussing recently proposed prevention strategies. Absence of guidelines results in a medical and surgical lack of expertise on how to approach these patients. This case is aimed at bridging a potential knowledge gap and emphasizing the importance of early surgical intervention as seen in prior reviews.

## 2. Case Description

A 66-year-old female with a history of gastric sleeve, paroxysmal atrial fibrillation on apixaban, status postradiofrequency ablation 4 weeks prior to presentation, hypertension, diabetes mellitus, and obesity, presented to an outside hospital with hematemesis and chills. Her vital signs were significant for the temperature of 103.1, and a chest CT with contrast revealed a left atrium to esophageal fistula. She had last taken apixaban the morning of admission. She was given prothrombin complex concentrate (PCC) and transferred to a tertiary center's cardiovascular intensive care unit (CVICU) for a higher level of care.

**Figure 1:**
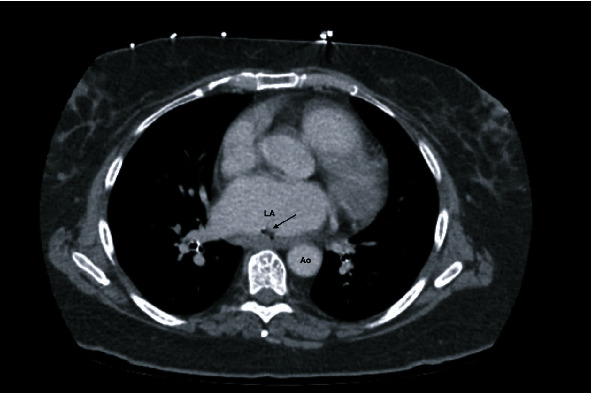
Cross-sectional image of CT scan with IV contrast demonstrating the atrio-esophageal defect. LA: left atrium; Ao: Aorta; arrow: extraluminal air adjacent to the esophagus.

**Figure 2:**
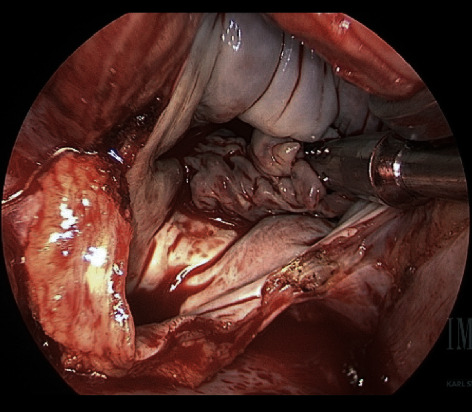
Left atrial vegetation.

**Figure 3:**
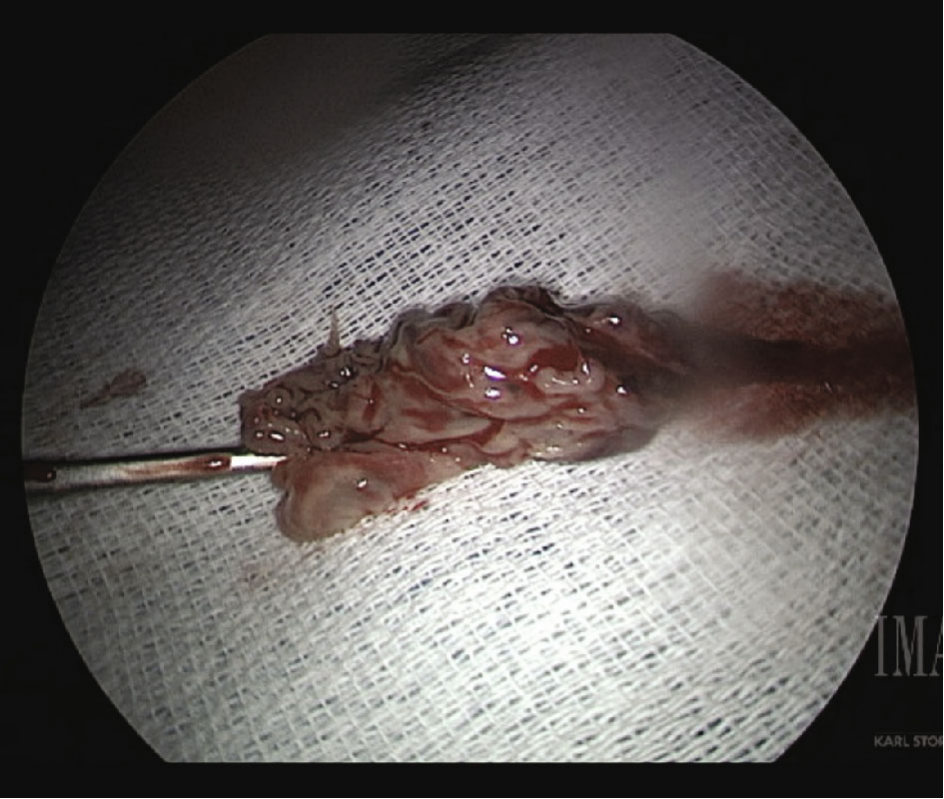
4 × 1.5 cm vegetation resected from the posterior left atrial wall.

**Figure 4:**
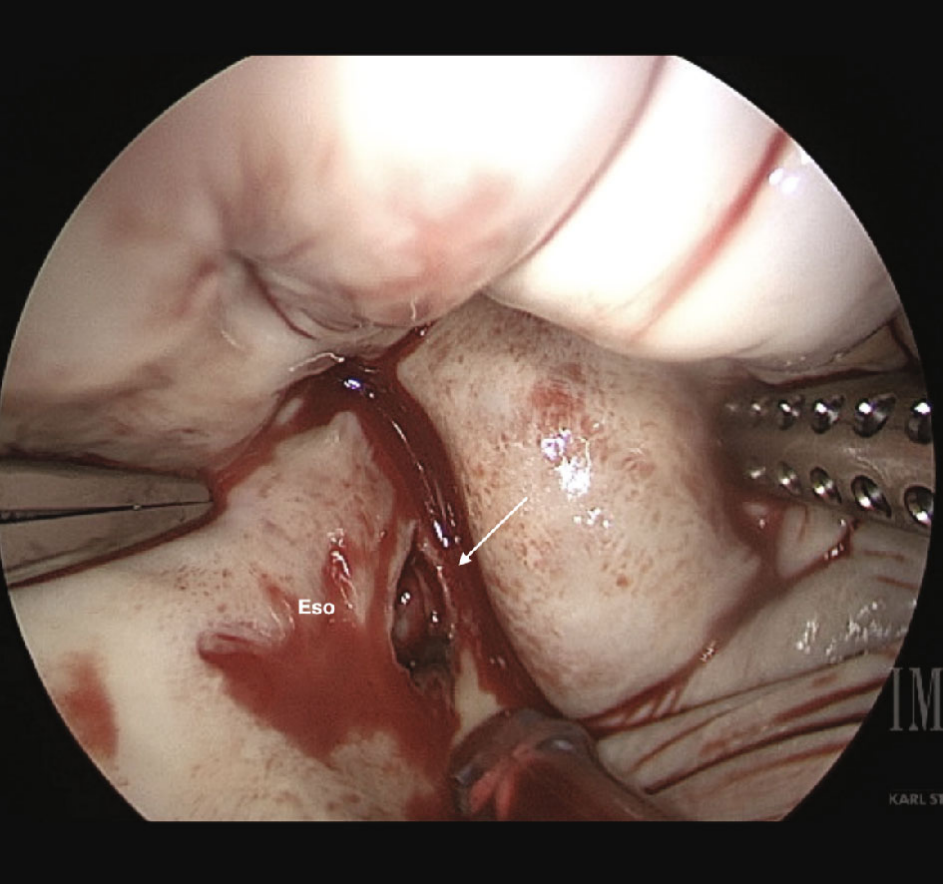
Esophageal injury. Eso: Esophagus. Arrow: 2 × 1.5 defect.

She arrived hemodynamically stable, with vital signs BP 102/50 mmHg, HR 85 on normal sinus rhythm, O2 saturation 97% on 2 L nasal cannula, and temperature 103 F. On review of systems, there was minimal nausea, denied chest pain, shortness of breath, and abdominal pain. A CT chest with contrast was repeated and redemonstrated intra-atrial air locule adherent to the posterior wall of the left atrium continuing through the wall and the posterior mediastinal paraesophageal soft tissues (Figure 1). Transthoracic echocardiography showed a small echogenic mass in the left atrium. Cardiothoracic surgery was consulted for surgical management. On hospital day 1, she had an episode of loss of consciousness with rigors concerning seizure activity; a CT head showed right occipital and left cerebellar hypodensities of unclear chronicity. Her outside blood cultures yielded *Streptococcus sanguinis* and broad spectrum coverage with fluconazole, vancomycin, and piperacillin/tazobactam was continued pending susceptibilities. The following day, she underwent primary closure of the left atrial defect. Intraoperative findings were significant for a 4 cm × 1.5 cm vegetation in the left atrium at the base of the endocardial defect (Figures 2 and 3). On hospital day 2, a repeat CT head showed multiple bilateral cerebral and cerebellar septic emboli. She underwent a right thoracotomy and closure of the esophageal and epicardial defect with an intercostal muscle flap (Figure 4). Her second surgery was complicated by pulseless ventricular tachycardia requiring defibrillation with immediate return of spontaneous circulation. She was supported in the ICU with epinephrine, amiodarone, and norepinephrine for mixed septic and cardiogenic shock and remained on mechanical ventilation for 6 days. Eventually, she underwent a J-tube placement. Intraoperative microbiology yielded methicilin-sensitive *Staphylococcus aureus* and alpha-hemolytic streptococci, and the antibiotic therapy was deescalated to Unasyn. Her neurologic exam was significant for dysphagia, delirium, and left-sided weakness. She was transferred out of the ICU on hospital day eleven and planned for six weeks of antibiotic therapy. A follow-up esophagram demonstrated no leak. She was discharged to a rehabilitation facility after an 18-day of hospital stay.

## 3. Discussion

Atrioesophageal fistula (AEF) is a rare complication of surgical or percutaneous ablation procedures. Although esophageal thermal lesions have been reported frequently, the incidence of complete atrio-esophageal fistula has been estimated at 0.01 to 0.04% based on surveys with variable participation [1–3], and mortality remains high among reported cases [4]. Given that the most common target of ablation procedures is the isolation of the pulmonary vein at the pulmonary vein-atrial junction, [5] the pathogenesis of AEF is linked to the anatomic relationship of the esophagus and left atrium, and microvascular damage with subsequent necrosis is the main injury mechanism. However, because AEF has a delayed presentation following the ablation procedure, acid reflux as a consequence of vagus nerve lesions has also been proposed. [6] In addition, contact force-sensing catheters might carry a higher risk of AEF formation [7].

### 3.1. Clinical Presentation

In a systematic review of 52 cases by Chavez et al., fever, neurologic deficits, and hematemesis were the most common presenting symptoms; [8] this correlates with findings described by another systematic review including 112 patients where the fever was present in 73% of cases [9]. The time to presentation was also similar in both reviews, averaging 21 days, but onset at two days and up to 60 days have been reported in the literature. [4, 10] Because fever and hematemesis can mimic other disease states, a high index of suspicion in the setting of a recent ablation procedure was key to pursue definitive diagnostic testing in this patient.

### 3.2. Diagnosis

Most authors agree that CT chest with IV contrast is the best diagnostic modality due to its low cost and high sensitivity [11]. Several radiologic features have been identified, with pneumomediastinum being the most common finding [9]. If AEF is suspected, avoiding esophageal manipulation (upper endoscopy and transesophageal echocardiography) is important to prevent increased esophageal pressure and air embolism. Other cases have reported the use of esophagram [9, 12]. However, in a patient presenting with neurologic symptoms, swallowing might be impaired, therefore precluding the use of an esophagram. CT head demonstrating cerebrovascular accidents in multiple vascular distributions are also suggestive of a cardioembolic source consistent with AEF. Transthoracic echocardiography (TTE), although not the modality of choice given the limitations in obtaining appropriate windows, was able to diagnose an AEF by visualizing air bubbles in the left ventricle during coughing episodes in a case report [13]. Although our patient had a nondiagnostic echogenic mass in the left atrium, the presence of fever, positive blood cultures, and cerebral embolic events were diagnostic of endocarditis by Duke's criteria as a complication of the fistulous tract.

### 3.3. Management

The prompt evaluation and referral to a higher level of care were keys to a successful outcome in this case. Once AEF has been identified, surgical intervention should be considered. A review by Yousuf et al. showed that mortality was 100% for patients managed with medical therapy or esophageal stenting alone, including the ones who died before planned surgical intervention [14]. A multivariate analysis by Han et al. confirmed that those undergoing conservative management had significantly higher mortality compared with those undergoing surgery [9]. Therefore, we advocate for early surgical intervention even in unstable patients.

Reported surgical interventions vary in the literature, with most specialists choosing a pleural or intercostal muscle flap for esophageal repair during the index operation to repair the atrial defect with or without cardiopulmonary bypass or shortly after via thoracotomy. [15, 16] The optimal surgical approach is unknown, but given the significant challenges associated with a combined sternotomy and thoracotomy, a staged approach could be considered.

In a case series of five patients, Kim et al. reported a successful case of a massive defect with air leak managed with VA ECMO-induced ventricular fibrillation in the preoperative period to minimize the risk of embolism; this could be considered as a transitory measure in a center with ECMO capabilities if the operating room or surgical team are not immediately available [17], but this should not delay operative intervention. Intensive care management with a multidisciplinary team of consultants that included neurology, infectious disease, and cardiothoracic surgery was of the utmost importance. Our patient was successfully discharged from the CVICU after eleven days of antibiotics, hemodynamic, perioperative, and nutritional support.

### 3.4. Prevention

Routine esophageal temperature monitoring and periprocedural use of proton pump inhibitors are considered standard preventative measures in many centers. Unfortunately, the low incidence of this complication translates into the lack of high-quality randomized evidence for their efficacy in preventing injury.

The significant morbidity and mortality associated with atrio-esophageal fistulas have led to a growing interest in preventative methods. Different techniques have been identified in recent years, with esophageal cooling devices becoming an attractive cost-effective option. In a randomized comparison between an esophageal cooling device with standard temperature monitoring, the intervention group had fewer thermal injuries on endoscopic examinations at one week follow-up when the esophageal temperature was maintained at 4 degrees Celsius throughout the ablation procedure [18]. Other novel strategies include reduction in contact force and energy, mechanical deviation of the esophagus, cryotherapy, pulsed-field ablation, and laser balloon technology [19]. Implementation of one technique over another will likely be influenced by operator skill and center capabilities.

## 4. Conclusion

Given the widespread implementation of catheter ablation as an attractive therapeutic option performed for more indications at more centers with different energy forms, it is likely that atrio-esophageal fistulas will occur in some cases. High-clinical suspicion of patients presenting with fever, hematemesis, and neurologic symptoms up to two months after AF ablation is important for survival.
